# COMBO-FISH Enables High Precision Localization Microscopy as a Prerequisite for Nanostructure Analysis of Genome Loci

**DOI:** 10.3390/ijms11104094

**Published:** 2010-10-21

**Authors:** Patrick Müller, Eberhard Schmitt, Anette Jacob, Jörg Hoheisel, Rainer Kaufmann, Christoph Cremer, Michael Hausmann

**Affiliations:** 1 Kirchhoff-Institute for Physics, Im Neuenheimer Feld 227, 69120 Heidelberg, Germany; E-Mails: muellerp@kip.uni-heidelberg.de (P.M.); eschmitt@kip.uni-heidelberg.de (E.S.); kaufmann@kip.uni-heidelberg.de (R.K.); cremer@kip.uni-heidelberg.de (C.C.); 2 German Cancer Research Center (DKFZ), Im Neuenheimer Feld 580, 69120 Heidelberg, Germany; E-Mails: a.jacob@dkfz.de (A.J.); j.hoheisel@dkfz-heidelberg.de (J.H.)

**Keywords:** COMBO-FISH, combinatorial oligo fluorescence *in situ* hybridization, computer based probe selection, PNA, SPDM, spectral precision distance/position determination microscopy, localization microscopy, nanostructure analysis of the genome

## Abstract

With the completeness of genome databases, it has become possible to develop a novel FISH (Fluorescence *in Situ* Hybridization) technique called COMBO-FISH (COMBinatorial Oligo FISH). In contrast to other FISH techniques, COMBO-FISH makes use of a bioinformatics approach for probe set design. By means of computer genome database searching, several oligonucleotide stretches of typical lengths of 15–30 nucleotides are selected in such a way that all uniquely colocalize at the given genome target. The probes applied here were Peptide Nucleic Acids (PNAs)—synthetic DNA analogues with a neutral backbone—which were synthesized under high purity conditions. For a probe repetitively highlighted in centromere 9, PNAs labeled with different dyes were tested, among which Alexa 488^®^ showed reversible photobleaching (blinking between dark and bright state) a prerequisite for the application of SPDM (Spectral Precision Distance/Position Determination Microscopy) a novel technique of high resolution fluorescence localization microscopy. Although COMBO-FISH labeled cell nuclei under SPDM conditions sometimes revealed fluorescent background, the specific locus was clearly discriminated by the signal intensity and the resulting localization accuracy in the range of 10–20 nm for a detected oligonucleotide stretch. The results indicate that COMBO-FISH probes with blinking dyes are well suited for SPDM, which will open new perspectives on molecular nanostructural analysis of the genome.

## 1. Introduction

The introduction of Fluorescence *in Situ* Hybridization (FISH) almost about 30 years ago marked the beginning of a new era in life sciences for the study of chromosome architecture and genome function. Nowadays, FISH has become a routine technique with a broad spectrum of commercially available probe kits optimized for biomedical research and diagnostics. The principle of FISH consists in hybridizing a fluorescently labeled nucleic acid probe completely to its complementary sequence in cell nuclei or on metaphase spreads. Probes with the respective targets are visualized at the single-cell level. During the last decades, FISH has been improved in sensitivity and specificity. Together with the advances in the fields of fluorescence microscopy and digital imaging resolution has also been enhanced. This progress has led to a better understanding of chromatin properties [1].

With further improvements of fluorescence light microscopy towards molecular resolution, e.g., STED–microscopy [2] or novel techniques of localization microscopy [3,4], investigations of the nanostructure of chromatin have become feasible [5]. At this level of precision, small but still target specific DNA probes (COMBO-FISH probes) that do not considerably influence the native nanostructure have been preferred for the analysis of individual gene targets [6].

COMBO-FISH [7,8] is a novel technique that allows precise and focused fluorescence labeling of chromatin domains in cell nuclei by computer selected combinations of short fluorescently labeled DNA or PNA probes (typically about 20–30 oligomers of about 15–30 nucleotides in length) [9,10]. Such a colocalizing probe set hybridizes in a defined genome region and causes a locus-specific fluorescence signal. Probe sets for double-helical or for triple-helical hybridization can be designed [8]. In order to obtain a specific label of a given chromatin target with short oligonucleotides, it is necessary to first identify candidate target sites and second to test these for reoccurrences against the complete human genome database by means of bioinformatic investigations [7,8,10,11]. By this means only those target sites are selected for a given gene locus that specifically colocalize at this region of interest, *i.e.*, the individual target sites may occur at several loci in the whole genome; however, *all* selected target sites only occur conjointly at the given genome locus. Finally, the resulting oligonucleotide probe sets can be synthesized. In contrast to standard FISH, for instance, with BAC (Bacterial Artificial Chromosome) clones, the small size of COMBO-FISH oligonucleotide probes should reduce structural alterations of the labeled chromatin target so that chromatin micro- and nano-architecture can be investigated under very mild conditions. In addition, using PNA oligonucleotides instead of DNA oligonucleotides has further advantages: (a) In contrast to DNA probes, PNA probes have a neutral backbone avoiding repulsive electrostatic forces from the negatively charged DNA target. This improves binding stability. (b) PNA probes open the application of COMBO-FISH to *in vivo* labeling since they are not enzymatically digested.

Thus, COMBO–FISH has several advantages in comparison to standard FISH: (A) Due to the theoretical probe design from the human genome database, any site can be precisely targeted and specifically labeled. (B) Denaturation of the double strand chromatin target can be omitted, which may allow specific chromatin domain labeling even of vital cells [12], which can further be improved by the use of PNA probes. (C) The entire sequence length of a probe set used for specific labeling is very small compared to the length of a gene target. For instance, for the ABL gene region on chromosome 9, only 31 oligonucleotide stretches with a total of 606 nucleotides label the 186,000 target nucleotides. Together, these oligonucleotide probes carry at the utmost 62 fluorochrome molecules [7]. This should considerably reduce any effects that the probe incorporation has on a genome domain’s spatial arrangement.

In a first proof of feasibility, we combined two novel techniques: gene size measurements by SMI (Spatially Modulated Illumination) microscopy [13] and COMBO–FISH, for size measurements of the ABL gene in 3D conserved blood cell nuclei [14]. In the application described in the following part of the article, we extend COMBO-FISH developments to applications of SPDM (Spectral Precision Distance/Position Determination Microscopy). SPDM is based on the fundamental concept of labeling objects by different spectral signatures or by using fluorophores that can be switched between two different spectral states to achieve a temporal isolation and thus a spatial separation of single signals. This allows the determination of object positions and distances even if they are very close together (<Abbe-Rayleigh limit of optical resolution). All acquired positions of fluorescent molecules can be merged into one image, in which the effective resolution is finally dependent only on the lateral and axial localization accuracy. SPDM localization microscopy has been improved into the precision range of 10 nm still using conventional fluorophores (e.g., fluorescent proteins or certain Alexa dyes) which are switched to a “dark” state by a light induced reversible photo bleaching.

Using PNA oligonucleotide probes [17] of a purine–pyrimidine mixed sequence with a PHYMOD (Physically Modifiable) fluorochrome (Alexa 488^®^–Invitrogen, Carlsbad, CA, USA) [15], here we show that individual dye molecules specifically labeling centromere 9 can be visualized and localized with a precision in the 10–20 nm range. Based on this breakthrough, COMBO-FISH and SPDM may open new insights into the nanoarchitecture of chromatin in gene targets.

## 2. Results and Discussion

### 2.1. PNA Probes

PNAs were synthesized and purified in parallel in microwell plates for a series of target sequences (for details see [18]). This process has advantages in those cases where small amounts of probe material but a high diversity of probe types under high quality and purity conditions are required. For the experiments presented in this article here, we tested several samples of the same purine–pyrimidine mixed PNA sequence with respect to sensitivity and labeling quality of different dyes. MALDI-TOF analysis of the purified labeled products revealed high quality results. The PNA sequences with the respective fluorochromes are listed in Table 1. Based on the concentration value, the probe volume for COMBO-FISH per slide was determined.

### 2.2. Microscopy Results

**Figure 1:**
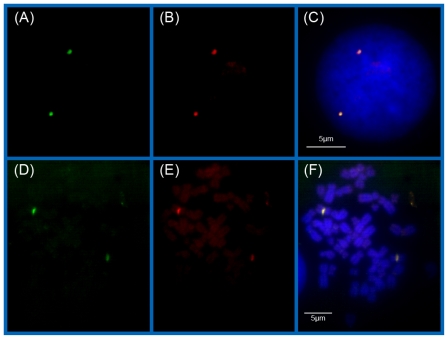
Examples of a lymphocyte cell nucleus (**A**–**C**) and a metaphase spread (**D**–**F**) after simultaneous labeling of the centromeric region of chromosome 9 with PNA COMBO-FISH probes. (**A**, **D**) green image plane: OregonGreen 488^®^ (λ_ex/em_ = 496/524 nm). (**B**, **E**) red image plane: TexasRed^®^ (λ_ex/em_ = 595/615 nm). (**C**, **F**) The merged images indicate a correct colocalization of the PNA signals. In addition, TOPRO-3-iodide was used for DNA counterstaining.

For the investigation described here, new type PNA oligonucleotide probes were used which were synthesized and purified according to new protocols. In order to test the hybridization quality of the PNA COMBO-FISH experiments, the three probe types were separately hybridized on methanol/acetic acid fixed peripheral blood lymphocyte cell nuclei and metaphase spreads. In all cases, the cell nuclei showed two fluorescent spots and the centromeres of two middle sized metaphase chromosomes were labeled (data not shown). To better verify the binding specificity and efficiency, we also performed simultaneous two color experiments with different spectrally labeled centromere 9 PNA probe types (one with OregonGreen 488^®^, the other with TexasRed^®^–Invitrogen, Carlsbad, CA, USA). Merging the green and red color image plane of the same nuclei or metaphases revealed a correct colocalization of the green and red labeling spot indicating competitive binding at the same target site (Figure 1). This seriously supports the centromeric specificity of the probe and reproducibility of the preparation procedure.

**Figure 2:**
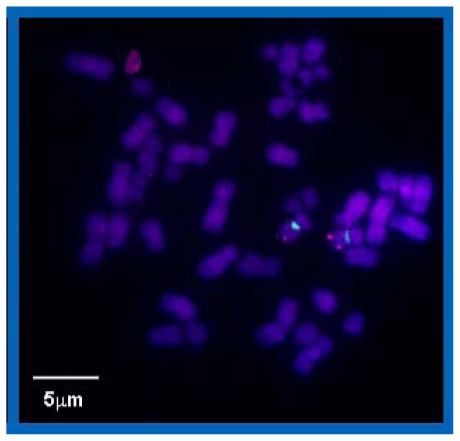
Example of a lymphocyte metaphase spread after a two-color experiment with the repetitive centromere 9 PNA COMBO-FISH probe (green signals: OregonGreen 488^®^, λ_ex/em_ = 496/524 nm) and a 9q subtelomere (9qtel) DNA standard FISH probe (red signals: rhodamine, λ_ex/em_ = 546/580 nm). For DNA counterstaining TOPRO-3-iodide was used.

In the next step, it was verified that the labeled chromosome is really chromosome 9. For this purpose, the lymphocyte specimens were subjected to another two-color experiment (Figure 2) using the repetitive centromere 9 PNA COMBO-FISH probe labeled with OregonGreen 488^®^ and a commercially available 9q subtelomere (9qtel) DNA standard FISH probe (MPbiomedicals, Montreal, Quebec, Canada) labeled with rhodamine. In these experiments, the metaphase spreads showed two chromosomes with red spots indicating the DNA probe marking the subtelomere ends of the long arms of human chromosome 9. The same chromosome carries the green signals of the PNA probes in the centromere region. This demonstrates the specificity of our synthesized PNAs applied here for chromosome 9.

### 2.3. Localization Microscopy

**Figure 3:**
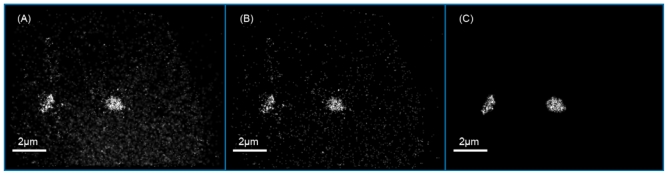
Examples of image sections of a mammary epithelial cell nucleus after COMBO-FISH of the centromeric region of chromosome 9 with repetitive PNA probes labeled with Alexa 488^®^ (λ_ex/em_ = 495/519 nm). Two labeling clusters of individual probe molecules are visible (**A**) Localization image of all detected signals. (**B**) Detected signals with a localization accuracy better than 30 nm. (**C**) Localization image of those signal molecules that have more than 40 blinking neighbors within a circle of 200 nm radius.

By illuminating the sample with a high laser excitation intensity (ten to several hundred kW/cm^2^ Alexa 488^®^ shows the characteristics of a PHYMOD (Physically Modifiable) dye, *i.e.*, a certain amount of molecules can be transferred to a reversibly bleached state (M_fl_ → M_rbl_). Stochastic recovery of these molecules to the fluorescent state (M_fl_ ← M_rbl_) allows for optical isolation of the detected single molecule signals during acquisition of a time stack of several hundred images of the same specimen region. Therefore we tested PNA COMBO-FISH labeling with Alexa 488^®^ for principle applications of SPDM using formaldehyde fixed, human mammary epithelial cells of the line AG11132 growing directly on coverslips (Figure 3).

**Figure 4:**
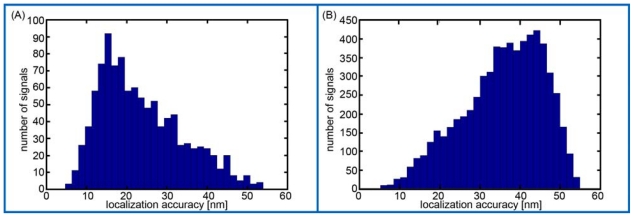
Frequency histograms of the localization accuracy of signals that have been detected within the (**A**) centromere clusters and (**B**) background region of the cell nucleus in Figure 3.

High sensitive SPDM of cell nuclei after COMBO-FISH revealed localization images in the green image plane with PNA probe signals and unspecific background presumably due to label-free fluorescence [19] or some unspecific probe attachment (Figure 3A). The effective optical resolution of this image was given by the density of detected signals and the localization accuracy. Therefore, the frequencies of localization accuracy values were determined for all signals registered within the centromere clusters and the background region (Figure 4). The majority of the signals referring to the centromere clusters revealed a localization accuracy better than 30 nm whereas the majority of background signals showed a worse localization accuracy. Thus, the centromere clusters were discriminated by a localization accuracy threshold of 30 nm (Figure 3B). Moreover it was shown that within these centromere clusters most signals had more than 40 neighboring signals within a circle of 200 nm radius (Figure 3C).

## 3. Experimental Section

### 3.1. Cell Culture and Specimen Preparation

For the experiments, two types of cells were used: (a) peripheral blood lymphocytes from a healthy donor; (b) cells of the line AG11132 (Coriell Institute, Camden, USA), human mammary epithelial cells of a healthy donor which have been established from normal tissue obtained at reduction mammoplasty.

Peripheral blood lymphocytes were isolated using a Ficoll gradient. 20 mL Ficoll (GE Healthcare, Munich, Germany) preheated to 37 °C were slowly covered with 30 mL heparinized whole blood. By means of centrifugation (200 g, 20 min) the cells were separated into four different gradients. The layer with the peripheral blood mononuclear cells was transferred into RPMI 1640/Fetal calf serum (FCS) (Invitrogen, Carlsbad, CA, USA/Biochrom, Berlin, Germany) (1:1, 37 °C). After centrifugation (200 g, 10 min) the supernatant was discarded. The pellet was washed once in 1 × PBS. The lymphocytes were further cultivated in preheated chromosome medium B (Biochrom, Berlin, Germany), which contains phytohaemagglutinin (PHA). After incubation for 72 h at 37 °C in a CO_2_-incubator, the medium was spiked with Colcemid (concentration 10 μg/mL) (Sigma-Aldrich, Hamburg, Germany) and the cell suspension was again incubated for 10–30 min at 37 °C. After centrifugation (200 g, 10 min) the supernatant was discarded. Then the hypotonic treatment followed with pre-warmed 75 mM KCl (Sigma-Aldrich, Hamburg, Germany). After incubation for 15 min at 37 °C and another centrifugation (200 g, 10 min) the supernatant was removed. The cells were then fixed by adding slowly −20 °C cold methanol/acetic acid (3:1) while the tube was agitated slightly. This fixation step was repeated 3–4 times.

AG11132 cells were grown in mammary epithelial growth medium (MEGM) supplemented with 4 μL/mL bovine pituitary extract, 5 μg/mL insulin, 5 ng/mL epidermal growth factor, 0.5 μg/mL hydrocortisone (MEGM BulletKit from Lonza, Walkersville, USA), 10^−5^ M isoproterenol (Sigma-Aldrich, Hamburg, Germany), 5 μg/mL transferring (Sigma-Aldrich, Hamburg, Germany), 10 mM HEPES buffer (Sigma-Aldrich, Hamburg, Germany) in a standard CO_2_-incubator. All cells were seeded onto glass slides (Menzel-Gläser, Braunschweig, Germany) and allowed to attach and grown overnight. Cells were fixed with 4% formaldehyde in PBS when they reached 80% of confluency. At all following steps (see below) drying of the formaldehyde fixed cells was avoided.

### 3.2. Design and Synthesis of COMBO-FISH Probes

The basics of database searching for appropriate combinations of oligonucleotides have been described in detail elsewhere [7]. Due to the completion of the human genome sequencing and the unification of the NCBI chromosome data files, the scope of search has been extended and the algorithms have been improved considerably. Using the annotation information for the region to be labeled from the corresponding NCBI contig files, the nucleotide sequences mapping these regions are identified and all homopurine and homoyrimidine stretches with a minimum length of 15 nucleotides are extracted by a straightforward search algorithm. This list of candidate individual target sequences is inspected for unfavorable stretches, e.g., with largely differing melting points or with periodicities, and for long sequences (more than 30 bases) which can be cut down to one or more shorter ones being more effectively synthesizable. The resulting ‘basic’ set is further processed to eliminate those oligonucleotide probes that form clusters of more than five stretches within any 250 kb genomic region elsewhere other in the genome than the selected target regions. For this purpose, the occurrence within the whole genome of all members of the basic set is listed. We prefer exact search to BLAST-search for three reasons: Firstly, gaps do not play any role at all, because the sequences have to fit exactly for binding. Secondly, we have to know securely each single occurrence of any stretch. And thirdly, nucleotide mismatches would lower the binding affinity, hence rendering non-exact matches to subordinate importance. It should be emphasized that, as purines pair to pyrimidines, it is necessary and sufficient to list both directions of one type of homosequences from the annotated strand. The finite automaton procedures for exact search are programmed in C. In the last semi-automated step, those candidate sequences, which occur frequently in clusters with more than five stretches within 250 kb, are iteratively removed until no further clusters except the labeling sites are left. For the proof of concept and feasibility studies presented here, the search was modified to purine-pyrimidine mixed probes. Here, an oligonucleotide (AAT CAA CCC GAG TGC AAT) has been chosen that uniquely exists as a repetitive sequence in the centromere region of chromosome 9 (see also [20]).

Only small quantities but high quality and purity of PNA sequences were needed. Therefore the PNA probes carrying different types of dye molecules (OregonGreen 488^®^, TexasRed^®^, Alexa 488^®^) were synthesized in 384 microwell plates followed by a low-cost parallel purification. Purity and recovery of the PNAs were determined by MALDI-TOF mass spectrometry and HPLC. Details will be published elsewhere [18].

### 3.3. COMBO-FISH

10–15 μL of the lymphocyte-solution was dropped on a wet slide. Slides were then air-dried. The pre-treatment of methanol/acetic acid fixed cells included multiple steps such as 0.7% Triton X-100/0.1% Saponin (Merck, Darmstadt, Germany/Serva, Heidelberg, Germany) in 2 × SSC for 30 min at room temperature, RNase A digestion (Sigma-Aldrich, Hamburg, Germany) (100 μg/mL in 10 mM Tris HCl, 1 h at 37 °C), pepsin-treatment (Sigma-Aldrich, Hamburg, Germany) (10% pepsin with a concentration of 100 mg/mL was diluted in a 0.01 M HCl, pH 2.3), dehydration of specimen in an aqueous ethanol series of 70%, 90%, and 100%. Denaturation was carried out in pre-warmed 70% formamide (AppliChem, Darmstadt, Germany) in 2 × SSC (pH 7.0) for 5 min at 75 °C. After denaturation, the slides were transferred back into an ice-cold ethanol series. Slides were air-dried.

The pre-treatment of formaldehyde fixed AG11132 cells included multiple steps such as 0.5% Triton X-100 in PBS, 20% glycerol (Sigma-Aldrich, Hamburg, Germany) in PBS, repeated freezing-thawing in liquid nitrogen and incubation in 0.1 N HCl. Slides were subsequently stored in 50% formamide in 2 × SSC (pH 7.0) at 4 °C until usage. Denaturation was carried out in pre-warmed 70% formamide in 2 × SSC (pH 7.0) for 3 min at 72 °C. After denaturation, the slides were transferred back into the 50% formamide in 2 × SSC (pH 7.0). The following steps (hybridization, washing steps and counterstaining) were accomplished equally for both preparation methods.

For hybridization, about 200 pmol/μL of the fluorescence labeled centromere 9 PNA probe (Table 1) was added to 20 μL hybridization buffer (0.6 M MgCl_2_, 3 M NaCl, 0.5 M sodium acetate, pH 7.0; Sigma-Aldrich, Hamburg, Germany). The mixture was applied onto the slide and covered with a cover slip, carefully sealed with rubber cement. Hybridization was done overnight at 37 °C in a humidity chamber. The next day, the rubber cement was removed and the slides were washed in 2 × SSC for 5 min at 42 °C. This step was repeated twice followed by another stringent washing step in 0.5 × SSC at 60 °C. The specimen was counterstained with TOPRO-3-Iodide (Invitrogen, Carlsbad, CA, USA) (1:1000) for 45 s, followed by washing three times in 1 × PBS and embedding in ProLong^®^ Gold antifade reagent (Invitrogen, Carlsbad, CA, USA). Finally the slides were covered with a coverslip (no. 1.5, 170 mm thickness, Menzel-Gläser, Braunschweig, Germany) and sealed with rubber cement (Marabu, Tamm, Germany) for microscopy.

### 3.4. Microscopy and Data Evaluation

For the acquisition of high resolution fluorescence images, an Ultra-View confocal spinning disc (PerkinElmer Life Science) on a Nikon TE2000-E inverted microscope was used (Nikon Imaging Center, Bioquant, Heidelberg), which was equipped with a Plan Apo 100x/NA1.4 oil immersion objective lens and lasers for excitation wavelengths of 488, 568 and 647 nm. The fluorescence was detected via appropriate band pass filters on an electron-multiplying charged-coupled device camera (EM-CCD, C9100-50, Hamamatsu). Emission of OregonGreen 488^®^ was detected via band pass filter 527 (55) nm, TexasRed^®^ via 625 (70) nm emission filter and TOPRO-3-iodide via 705 (90) nm. The lateral scanning area was up to 1000 × 1000 pixels corresponding to a voxel size laterally of 82 nm × 82 nm. Within this scanning area, a ROI (region of interest) was selected according to the size of the cell nucleus recorded. The step width for optical sectioning was 200 nm in axial direction. All the 3D image stacks of the cell nuclei were evaluated by Matlab based algorithms.

Localization measurements were performed with the SPDM setup [4]. Optical isolation of signals was achieved utilizing a light induced reversibly bleached state [21–23] of Alexa 488^®^. By illuminating the sample with a laser excitation intensity of 10 kW/cm^2^ to several 100 kW/cm^2^ some fluorophores are bleached irreversibly (M_fl_ → M_ibl_), but another amount is transferred to a reversibly bleached state (M_fl_ → M_rbl_). Stochastic recovery of these molecules to the fluorescent state (M_fl_ ← M_rbl_) can be used for optical isolation of the detected single molecule signals. This method allows the usage of a series of conventional fluorophores (PHYMOD dyes) for localization microscopy.

For the present experiments, a DPSS laser with a wavelength of 488 nm (Sapphire HP 488, Coherent, Dieburg, Germany) was used. The laser beam was expanded by a factor of 2.5 before being focused into the back focal plane of an oil immersion objective lens (HCX PL APO, 63x, NA = 0.7–1.4, Leica, Wetzlar, Germany). Fluorescent light emitted by the fluorophores in the sample passed a dichroic mirror (AHF Analysetechnik, Tübingen, Germany) and a blocking filter (F73-491, AHF Analysentechnik, Tübingen, Germany) before being focused onto the CCD chip of a very sensitive camera (SensiCam QE, PCO Imaging, Kehlheim, Germany). An additional lens can be mounted in the excitation pathway for increasing the laser intensity in the object plane to obtain appropriate conditions for the reversible photo bleaching.

For the measurements presented here, data stacks consisting of several thousand images were recorded with an integration time of the camera of 50 to 150 ms. After conversion of the count numbers into photon numbers, a differential image stack was calculated by subtracting the succeeding from the preceding frame. This had to be done to filter out the signals of the single molecules due to background noise and bleaching gradients of biological samples. After segmentation of the raw data, a 2D Gaussian was fitted to the single molecule signals to determine the positions of the detected molecules [4,15,16,24]. Using this information, a localization image was rendered by blurring the position of each detected molecule with a Gaussian corresponding to the individual localization accuracy.

## 4. Conclusions

During the last 30 years, FISH has been developed into a powerful tool in biological research and medical diagnostics. For the first time, it has become possible to investigate the structure–function correlation of the genome, the architecture of chromosomes, and subchromosomal domains in intact cell nuclei. With the improvement of fluorescent microscopy and novel techniques circumventing the Abbe-Rayleigh diffraction limited resolution conditions, science has entered the cosmos of genome nanostructure analysis under native conditions. This has forced methodologists to further promote FISH to the application of nanoprobes on the level of oligonucleotides still maintaining the high specificity of standard FISH probes.

COMBO-FISH fulfils many requirements of nanostructure labeling for fluorescent light nanoscopy like localization microscopy. A combinatorial approach of probe set design maintains specificity by unique colocalization of oligonucleotides while only small DNA/PNA–stretches with one or two fluorochromes each are used for hybridization. This avoids harsh treatment and should reduce structure modifications on the nano-level of gene targets to a minimum.

Here, we present for the first time evidence that PNAs synthesized in small scales with high purity, labeled with PHYMOD dyes like Alexa 488^®^ are well suited for SPDM. Although our data only demonstrate the principle feasibility of COMBO-FISH SPDM, the data clearly show that real COMBO-FISH labels can be discriminated from background signals by localization accuracy. Moreover, the detection of clusters of single molecule signals indicates that it should be possible to quantitatively analyze nanostructures of chromatin within the domains of genes or gene subregions. This will open new perspectives in the investigations of structure dependent genome function or dysfunction.

## Figures and Tables

**Table 1:** PNAs labeled with different dyes (with excitation λ_ex_ and emission λ_em_ maxima) and their amounts used for specimens in this article. All probes were specific for centromere 9.

PNAs with fluorochrome	λ_ex/em_ [nm]	Probe concentration	Probe amount used for COMBO-FISH
PNA sequence with Alexa 488^®^	495/519	162.3 pmol/μL	1.3 μL
PNA sequence with OregonGreen 488^®^	496/524	203.9 pmol/μL	1.0 μL
PNA sequence with TexasRed^®^	595/615	94.3 pmol/μL	2.0 μ
